# A study on oxidative stress and antioxidant status of agricultural workers exposed to organophosphorus insecticides during spraying

**DOI:** 10.4103/0019-5278.58916

**Published:** 2009-12

**Authors:** S. K. Rastogi, P. V. V. Satyanarayan, D. Ravishankar, Sachin Tripathi

**Affiliations:** Department of Indian Institute of Toxicology Research, Lucknow, UP, India; 1 Biochemistry, Acharya Nagarajuna University, Guntur, AP, India

**Keywords:** Acetylcholinesterase, organophosphorus insecticides pesticides, oxidative stress

## Abstract

Oxidative stress status and Acetylcholinesterase (AChE) activity were studied in blood samples obtained from 61 agricultural workers engaged in spraying organophosphorus (OP) insecticides in the mango plantation, with a minimum work history of one year, in the age range of 12-55 years. Controls were age-matched, unexposed workers, who never had any exposure to OP pesticides. They were evaluated for oxidative stress markers MDA (end product of lipid peroxidation), reduced glutathione (GSH), and Acetylcholinesterase (AChE) and butyrylcholinesterase (BChE) levels in blood. The results showed a marked inhibition of the AChE and BChE activities in the sprayers as compared to the controls. The malondialdehyde(MDA), the last product of lipid peroxidation was found to be increased significantly in sprayers(p<0.05), while depletion in the concentration of antioxidant glutathione(GSH) was also observed in the sprayers but the difference was statistically not significant. It was concluded on the basis of biochemical analysis that pesticides sprayers are exposed to more oxidative stress as evidenced by the changes in antioxidant status. The measurement of the AChE and BChE activities in agricultural workers who spray OPs could be a good biomonitoring factor and is recommended to be performed on a regular basis.

## INTRODUCTION

For centuries, several hundred pesticides have been used in agricultural practice in order to enhance food production by eradicating unwanted insects and controlling disease vectors. These pesticides differ greatly in their modes of action, uptake by the body, metabolism, elimination from the body, and toxicity to humans.[1] Among the pesticides organophosphorus insecticides (OPs) and carbamates have been widely used, as these compounds are non-persistent in the environment.[2] OPs are frequently sprayed in mango plantations of Malihabad, Lucknow, (U.P.), which is a mango belt in north India, and remain an important source of poisoning.[3] Occupational exposures to these pesticides occur from skin absorption and inhalation, as the sprayers are from poor families and are not in the habit of using face masks and other protective devices.[4] The exposure of pesticides mainly occurs in the mixing and loading of equipment, in the spraying of insecticides, and improper handling. There are strong associations between exposure to aerial pesticides and neurological symptoms, as the cholinesterase activity is significantly reduced in the exposed pesticide sprayers.[3,4] Cases of chronic neurotoxicity among farm workers exposed to organophosphorus insecticides have been reported in the literature.[5–10] Most OP insecticides exert their toxicity on the target and non-target organs through inhibition of acetylcholinesterase in the nerve and muscle tissues.[11–13] Actual exposures to insecticides can be assessed by the biological monitoring of human tissues and body fluids.[14]

The most economical blood test for the monitoring of farm workers who are exposed to organophosphorus insecticide is the plasma butyryl cholinesterase activity, its inhibition is taken as a biomarker for exposure.[15,16] Monitoring of plasma butyrylcholinesterase (PbChE) has been recommended in the OP-exposed population as this could be a useful biomarker to predict and prevent the health hazards of pesticides.[14] Toxicity of organophosphates causes adverse effects on different systems in the body, including hematological and biochemical changes.[17] More recently, the reactive oxygen species (ROS) have been implicated in the toxicity of the pesticides. Organophosphates induce changes characteristic of oxidative stress.[18–20] A significantly elevated MDA (end product of lipid peroxidation) level was observed in the sprayers exposed to OP, carbamate, and organochlorine pesticides, when compared to the controls,[21] suggesting that oxidative stress may be involved in the toxicity of pesticides.[22–23] Although studies are available on lipid peroxidation products and the antioxidant status in experimental animals, with an individual or group of pesticides,[21] no detailed report is available on subjects who apply different categories of OP pesticides. In a study from India, a significantly higher MDA level, with inhibited AChE activity of acute organophosphate-poisoned patients was found.[21–24] It is of interest to study the antioxidant status and the extent of oxidative stress in the agricultural workers engaged in spraying the organophosphorus insecticides in the mango plantation.

## MATERIALS AND METHODS

### Subjects

The study was conducted in the neighboring villages of Lucknow in North India during the period October to November 2008. The study included the total rural population of 225 sprayers (male 132/93 females), spraying a number of organophosphate pesticides in the mango plantations of Mal, Malihabad, and Rahimabad villages of Lucknow. A control group consisting of 50 unexposed workers, who never had any exposure to OP pesticides was taken as a reference group. The OP pesticides mainly sprayed were dichlorvos, methyl parathion, chlorpyrifos, and so on [Table 1].

**Table 1:** List of organophosphorus pesticides sprayed in mango orchards in the neighboring villages of Lucknow

Dichlorvos
Methyl parathion
Phorate
Chlorpyrifos methyl
Monocrotophos
Acephate
Diazinon
Malathion
Ethion
Chlorpyrifos
Quinalphos
Profenofos
Triazophos

A detailed history, including the personal and occupational history, was recorded in each case on a pre-structured survey proforma. Smokers and alcoholics were excluded from the study. The spray procedures and conditions of equipment, eating and drinking habits, and personal cleanliness were also recorded in the proforma. Mixing of all the pesticides with bare hands, leakage from the pesticide tank, and eating food without proper washing during and after spraying operations were found to be very common among all pesticide applicators. Following this the venous blood was collected in heparinized tubes and brought to the laboratory in an ice box for analysis of the following biochemical parameters.

### Chemicals and reagents

Thiobarbituric acid (TBA), Reduced glutathione (GSH), 5,5'-dithio-bis-(2-nitrobenzoic acid) (DTNB), and acetylthiocholine iodide were from the Sigma Chemical Company. All the other chemicals used were of the highest purity available from commercial sources.

### Biochemical parameters

The extent of lipid peroxidation in the whole blood was assayed by measuring the formation of thiobarbituric acid reactive substances (TBARS) using the method of Stocks *et al*., and expressed as nmol MDA formed per milliliter of blood, using a molar extinction coefficient of 1.56 × 10^5^ L/mol cm.[25] Reduced glutathione (GSH) was estimated in the whole blood using the Ellman's reagent and expressed as μg/ml of blood.[26] Blood Acetylcholinesterase (AChE) and butyrylcholinesterase (BChE) activity was determined by the method of Ellman *et al*.,[27] as modified by Chambers,[28] by taking acetylthiocholine iodide as the substrate and expressed as mmols hydrolyzed/h/l blood (I/U). The biochemical parameters were assessed only in 61 OP pesticide sprayers as others refused to give blood samples.

## RESULT

**Figure 1:**
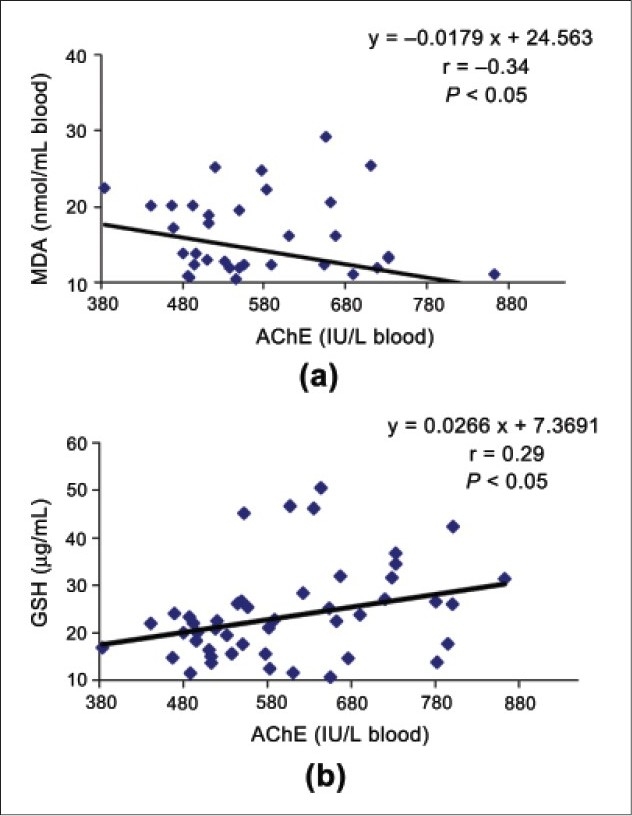
Significant correlation of biochemical parameters in the blood samples of subjects (n = 61).

The demographic features of both groups of subjects are shown in Table 2. The age, height, weight, body mass index (BMI), education, dietary habits, ethnicity, area of residence, and socioeconomic status of the subjects from both groups were almost similar. The work exposure of mango orchards sprayers was 1-18 years (average 15.94 ± 5.89 years). The biochemical profiles of both groups, that is, the sprayers and controls are shown in Table 3. The activities of the AChE and BChE enzymes were significantly lower in the sprayers as compared to the controls (*P* < 0.05). The blood MDA level, as an end product of lipid peroxidation, was significantly higher in the sprayers than those in the controls (*P* < 0.05). However, the decrease in GSH level, an antioxidant enzyme was not significant in sprayers. Figure 1 represents the correlation of the biochemical parameters in the blood samples. A significant correlation of blood AChE was found with MDA and GSH levels; blood AChE-blood MDA (r = 0.34, *P* < 0.05) and blood AChE-blood GSH (r = 0.29, *P* < 0.05).

**Table 2:** Comparison of demographic features between the pesticide sprayers and controls

Variables	Controls (n = 50) mean ± SD	Sprayers (n = 225) mean ± SD
Age (y)	28.40 ± 4.04 (19-31)	27.3 ± 9.37 (12-55)
Height (cm)	170 ± 6 (155-182)	157 ± 6 (143-169)
Weight (kg)	63.25 ± 11.25 (44-82)	47.9 ± 6.87 (44-71)
BMI (kg/m^2^)	22.21 ± 2.01 (17.8-28.7)	17.41 ± 2.01 (18.9-26.4)
Addiction		
Tobacco/smoking/alcohol		
Users	24 (48)	170 (75.55)
No-Users	26 (52)	55 (24.44)
Education		
Literate	50 (100)	90 (40)
Illiterate	0	135 (60)
Ethnicity		
Urban	0	0
Rural	50 (100)	225 (100)
Socioeconomic status		
High	0	0
Middle	28 (56)	153 (68)
Low	22 (44)	72 (32)
Religion		
Hindu	41 (82)	147 (65.33)
Muslim	9 (18)	78 (34.67)
Diet		
Vegetarian	28 (56)	141 (62.67)
Non-vegetarian	22 (44)	84 (37.33)
Experience of pesticide spraying (Y)	0	5.94 ± 5.89 (1-18 y)

**Table 3:** Biochemical profiles of sprayers and controls

Biochemical parameters	Controls (n = 18) mean ± SE	Sprayers (n = 61) mean ± SE
AChE (IU/l)	691 ± 19.2.6 (535.1-804.8)	585.2 ± 21.3* (405.3-795.9)
BChE (IU/l)	615.1 ± 39.2 (411.9-783.7)	484.4 ± 13.9* (298.4-602.4)
GSH (μg/ml)	295 ± 34.7 (149.6-541.84)	224.7 ± 19.7 (96.4-440.3)
MDA (nmol/ml)	11.3 ± 0.83 (6.32-18.48)	39.30 ± 3.07* (18.04-73.41)

* Parenthesis indicates the range *P* < 0.05

## DISCUSSION

The study indicated that there was a significant decrease in activity of AChE and BChE and an increased level of MDA found among the OP pesticide sprayers as compared to the controls. Our result supports the earlier findings of Vidyasagar *et al*.,[24] as they also found inhibited AChE activity in the OP-exposed workers. There was a significant correlation between AChE and GSH and AChE with MDA, thereby, indicating an enhanced lipid peroxidation in sprayers, with cholinesterase inhibition. The inhibition of AChE might have resulted in the accumulation of ACh at the synaptic junctions, leading to cytotoxity and increased lymphocyte motility.[29] The GSH level might be decreased due to its participation in the activation, inhibition, and progression of lymphocytes and increased GST activity.[29] Moreover, the decline in GSH level observed in the study could be attributed to the conjugation reactions (GSH consumption) superseding the cell ability, to regenerate GSH. The increased level of MDA in the present study confirms the earlier report.[21] As the sprayers predominantly developed neurotoxic symptoms on exposure to OP pesticides they developed acute OP poisoning, which corroborated with the clinical and biochemical findings reported in this study.

## ACKNOWLEDGMENTS

This study was supported by Council of Scientific and Industrial Research (CSIR) for providing the fund. The authors also thankful to Indian Institute of Toxicology Research, Lucknow (U.P.) India for providing the facilities doing this work.

## CONCLUSION

An increased level of MDA in exposed pesticide sprayers is probably reflective of increased lipid peroxidation and cell damage (Oxidative stress).
